# Brain Structure and Function: Insights from Chemical Neuroanatomy

**DOI:** 10.3390/life13040940

**Published:** 2023-04-03

**Authors:** Luigi F. Agnati, Diego Guidolin, Chiara Cervetto, Guido Maura, Manuela Marcoli

**Affiliations:** 1 Department of Biochemical, Metabolic Sciences and Neuroscience, University of Modena and Reggio Emilia, 41125 Modena, Italy; 2 Department of Neuroscience, University of Padova, 35121 Padova, Italy; diego.guidolin@unipd.it; 3 Department of Pharmacy, University of Genova, 16148 Genova, Italy; chiara.cervetto@unige.it (C.C.); guido.maura@unige.it (G.M.); 4 Center for Promotion of 3Rs in Teaching and Research (Centro 3R), 56122 Pisa, Italy; 5 Center of Excellence for Biomedical Research, University of Genova, 16132 Genova, Italy

**Keywords:** brain hyper-network, multi-level hierarchical, nested architecture, wiring transmission, volume transmission, allosteric interactions, receptor mosaics, penta-partite synapses

## Abstract

We present a brief historical and epistemological outline of investigations on the brain’s structure and functions. These investigations have mainly been based on the intermingling of chemical anatomy, new techniques in the field of microscopy and computer-assisted morphometric methods. This intermingling has enabled extraordinary investigations to be carried out on brain circuits, leading to the development of a new discipline: “brain connectomics”. This new approach has led to the characterization of the brain’s structure and function in physiological and pathological conditions, and to the development of new therapeutic strategies. In this context, the conceptual model of the brain as a hyper-network with a hierarchical, nested architecture, arranged in a “Russian doll” pattern, has been proposed. Our investigations focused on the main characteristics of the modes of communication between nodes at the various miniaturization levels, in order to describe the brain’s integrative actions. Special attention was paid to the nano-level, i.e., to the allosteric interactions among G protein-coupled receptors organized in receptor mosaics, as a promising field in which to obtain a new view of synaptic plasticity and to develop new, more selective drugs. The brain’s multi-level organization and the multi-faceted aspects of communication modes point to an emerging picture of the brain as a very peculiar system, in which continuous self-organization and remodeling take place under the action of external stimuli from the environment, from peripheral organs and from ongoing integrative actions.

## 1. General Premises

Most of the new evidence on the structure and functions of the brain is emblematic of the scientific advances made possible by synergic interactions among different disciplines. In particular, the synergic interactions between the approaches of biochemical anatomy and computer-assisted image analyses have produced a profoundly different view of brain structure and functions, hence also paving the way to possible new medical therapies and the proposal of formal models of brain integrative actions.

Indeed, in the last century, the extraordinary progress made in the investigations of the structure and functions of the brain were mainly due to the epistemological intermingling of chemical anatomy (i.e., histology, histochemistry, immunocytochemistry), new microscopy techniques (e.g., confocal and two–photon microscopy, electron microscopy, atomic force microscopy) and computer-assisted morphometric methods, allowing detailed quantitative analysis of the images yielded by these new techniques. In this conceptual paper, we discuss some crucial aspects of the theoretical and experimental contributions made by our group to the investigations of brain structure and functions.

The complex combination of different approaches has led to the proposal of new scientific “paradigms”. Not only can this different view attract researchers, but it is also sufficiently unrestrained to enable them to propose new approaches that can yield experimental evidence from a different perspective [1,2].

The two main researchers who opened up a “new multi-faceted paradigm” of brain structure and function were Golgi and Cajal, who made fundamental contributions to the investigation of the nervous system through their work in histology. Thus, the morphological features, location and possible network organization of the cells of the nervous system became a new field of investigation of brain integrative functions.

As mentioned above, a subsequent crucial step was the computer-assisted analysis of images yielded by various chemical approaches to neuroanatomy (see [3,4,5]). This was the turning point that allowed extraordinary investigations to be carried out on brain circuits, leading to the development of the new discipline of “brain connectomics” (ref. [6] and see below). This new approach enabled brain structure and function to be characterized in physiological and pathological conditions. Moreover, it prompted the development of new therapeutic approaches; these concerned not only pharmacological treatments but also surgical interventions and even attempts to overcome crucial deficits in the central nervous system (CNS) circuits by means of in situ electro-chemical stimulation [7,8] or transcranial photo-biomodulation [9].

A new pharmacological approach that exploits brain connectomics at the molecular level is based on signal integration at the plasma membrane level, specifically, on the allosteric modulations of receptors. Indeed, allosteric receptor modulators provide greater receptor subtype selectivity and temporal selectivity as a result of the release of the endogenous ligand [10,11], thus, they are currently being investigated for purposes of CNS therapy [12,13]. In this context, it has been shown that allosteric receptor–receptor interactions (RRIs) and druggable allosteric sites appear when protomers assemble into receptor heteromers, which are involved in the recognition and decoding of signals at the plasma membrane level (refs. [14,15,16,17,18,19], see also [20,21] for a review).

## 2. From the Contributions of Golgi and Cajal to Modern Chemical Neuroanatomy: A Brief Survey

First, it should be pointed out that Golgi (1843–1925) and Cajal (1852–1934) held different views of the morpho-functional organization of the brain. However, studies conducted in recent decades have revealed that their views were, in fact, highly complementary, rather than markedly different, as will be discussed below [22].

On the basis of his morphological studies of neurons visualized by means of “the black reaction” (a histological silver staining technique; for details see, e.g., [23]), Golgi hypothesized that continuity among neurons was provided by a network of branching and anastomosing nerve processes that formed a “diffuse nerve network” in all layers of the gray matter of the brain; hence, single neurons had no functional identity. In his view, inter-neuronal communications could occur by means of electrical signals, since the medium interposed between neurons is an electrolytic (hence electrically conductive) solution [24].

Cajal proposed a different view, the so-called “neuron doctrine”, according to which neurons were anatomically discrete and functionally independent units, which were interconnected via specialized regions of contiguity, and not of continuity [25].

Sherrington, on the basis of his experimental functional data, accepted Cajal’s proposal, as he clearly stated in his book [26]: “… *if at the nexus between neuron and neuron there does not exist actual confluence of the conductive part of one cell with the conductive part of the other, … there must be a surface of separation… vertebrate histology on the whole furnishes evidence that a surface of separation does exist… In view, therefore, of the probable importance physiologically of this mode of nexus between neuron and neuron it is convenient to have a term for it. The term introduced has been synapse*.”

Sherrington’s assumptions set the scene for the development of a new field of morpho-functional investigations on brain networks. Indeed, analyses focused on the synapse, which is a region of discontinuity (the synaptic cleft) between neurons, and how communication between pre-synaptic and post-synaptic neurons could occur via chemical and/or electrical signals across the synaptic cleft.

In sum, Cajal–Golgi mapping of the brain by means of the silver staining technique had important consequences: neuronal networks were described; the shapes of neurons were analyzed in detail; different classes of neurons were identified, as was their spatial distribution in brain areas; and differences in neuronal parameters were ascertained in different species [27]. However, especially in view of the neuron doctrine, this basic step was followed by several further steps.

In 1934, Dale hypothesized that the same chemical transmitter was released from all the synaptic terminals of a neuron [28,29,30,31]. The first electron microscope images of the axon terminals and synapse [32,33,34] led to the vesicular hypothesis of neurotransmission, whereby small synaptic vesicle discharge at the synapse is the basis for neurotransmission. A breakthrough occurred with the introduction of Falck and Hillarp’s formaldehyde monoamine fluorescence technique in 1962, whereby catecholamines and serotonin were converted into fluorescent compounds that could be visualized at the cellular level [35]. This new histochemical approach not only enabled monoamine neurons in the brain to be mapped [36,37,38,39,40], but also led to important progress in neuropsychopharmacology. These maps and functional data on the effects of monoamine on brain integrative actions had a greater impact when the computer-assisted image analysis of histochemical preparations was made possible [5,41,42,43]. A preliminary study of the post-synaptic side was carried out through the mapping of the receptors for transmitters in the brain by means of receptor autoradiography [44]; see also [4,41]. This was followed by the mapping of the transmitter-identified neuronal systems by means of histochemistry and immunohistochemistry [45,46].

A further important step forward was the demonstration that the original view of the synapse, as proposed according to Dale’s principle, was mistaken [47,48,49,50,51]. Indeed, several authors (see bibliography from [48,50,51,52,53,54]) demonstrated that one neuron could synthesize more than one neurotransmitter. Thus, Dale’s principle was superseded, as it stated that one neuron contained, and could release, only one neurotransmitter, which exerted the same effects at all synaptic connections [28,29,30,31,55].

In reality, more than one neurotransmitter can be released from the pre-synaptic site, and a single post-synaptic site may express different types and subtypes of receptors for a given transmitter, with each receptor controlling a different decoding mechanism or ionic conductance channel [56,57]. Thus, a synapse becomes endowed with multiple communication/transmission lines, each of which is represented by its own neurotransmitter, and each transmitter can be decoded by a set of different receptors [58]. Indeed, diverse processes of co-release of neurotransmitters have been identified (see [59]). Small classic molecule transmitters and neuropeptides, for instance, are usually packaged in separate vesicles, leading to different capacities in terms of vesicle mobilization, release and post-synaptic targets. Fast-acting neurotransmitters, by contrast, are often released from the same vesicle. In any case, the prevalence of multi-transmitter synapses indicates that these are of key importance to brain activity [60]. The spectrum of the diverse effects that they can exert can be explored by means of suitable electrophysiological methods (recently reviewed by Kim and Sabatini [59]). An interesting example is provided by studies on co-release involving GABA (see [61] for a review). The release of GABA combined with other co-transmitters (including glycine, glutamate, acetylcholine, dopamine and histamine) may endow synapses with high functional flexibility. The co-release of GABA with excitatory transmitters, for instance, may fine-tune the membrane potential of target cells. A second example concerns monoamines, which mainly signal through GPCRs. Thus, their synaptic effects are exerted in hundreds of milliseconds or more, while GABA co-release allows a temporally precise signal to be created and transmitted to sensory or motor systems [61].

**Figure 1:**
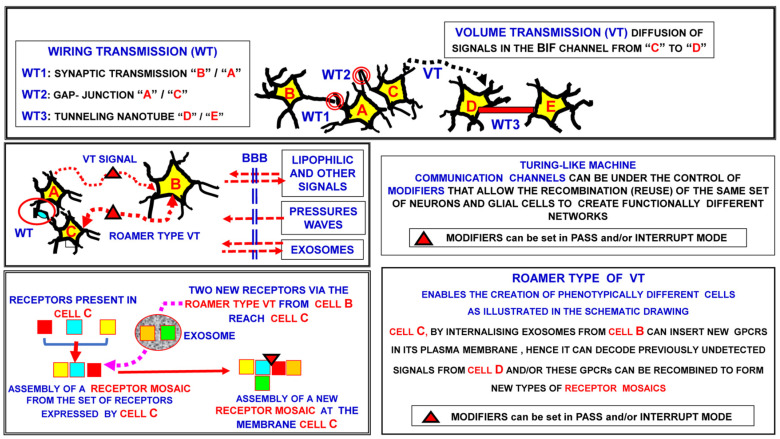
Schematic representation of intercellular communications in a neural network. While VT refers to the diffusion of signals, roamer type VT occurs as a result of the migration of extracellular vesicles into the extracellular space to reach the target cells. Hence, a basic feature of the VT communication mode, namely migration into the extracellular space, characterizes the roamer type VT communication mode. BBB: blood-brain barrier; BIF: brain interstitial fluid.

Furthermore, in some instances, even if the neuron synthesizes more than one neurotransmitter, there is a process of selection and segregation of different neurotransmitters at different pre-synaptic terminals, which face distinct excitatory or inhibitory post-synaptic sites. As discussed by Saunders and co-authors and by Cifuentes and Morales [55,62], segregation changes depend on the requirements of the network’s integrative function, and hence should be considered an important plastic property of neurons that is capable of increasing the neuronal signaling repertoire. Thus, the synaptic contacts are surprisingly highly plastic devices, not only from a structural point of view, but also in view of their functional ductility. This will be discussed further in the section dealing briefly with RRIs in the context of Changeux’s pioneering contribution on allostery (see [63] and the bibliography quoted therein). Figure 1 provides a schematic view of inter-neural communication and the possible importance of Turing’s unorganized machine to several communication modes. Some of these aspects will be briefly discussed in the following sections of the paper.

Although most of the histochemical studies focused mainly on neuronal cells, early investigations had already revealed the crucial roles played by the extracellular matrix networks (ref. [64,65] and glial cells in the functional and structural organization of the brain (ref. [66]; see [67] for a historic account). Their potential importance for the integrative functions is still a crucial area of investigation in brain physiology and pathology [67,68,69,70]. Indeed, the brain’s integrative actions are the results of inter-cellular communication processes, not only between neurons but also with other types of cells, and these processes are modulated by the structure and chemical composition of the extracellular matrix (refs. [71,72,73] and bibliography cited therein). Thus, investigation of the brain’s integrative functions should consider ‘complex cellular networks’, i.e., functional networks that include neurons, astrocytes, microglial cells, oligodendroglial cells, ependymal cells, pericytes and mast cells, and also the extracellular matrix [74]. Although the complex relationship between the glial cells and the neurons have been investigated by several groups [75,76], some aspects are still controversial; for example, even the question of glial cell counting has not been definitively settled (e.g., [77,78]). Our group has especially investigated a peculiar field of astrocyte function, i.e., the integrative mechanism of signal recognition and decoding at the plasma membrane level based on allosteric interactions among G protein-coupled receptors (GPCRs) (i.e., RRIs; see below) and their possible importance in neuropathology (see [20,70,79,80,81]).

**Figure 2:**
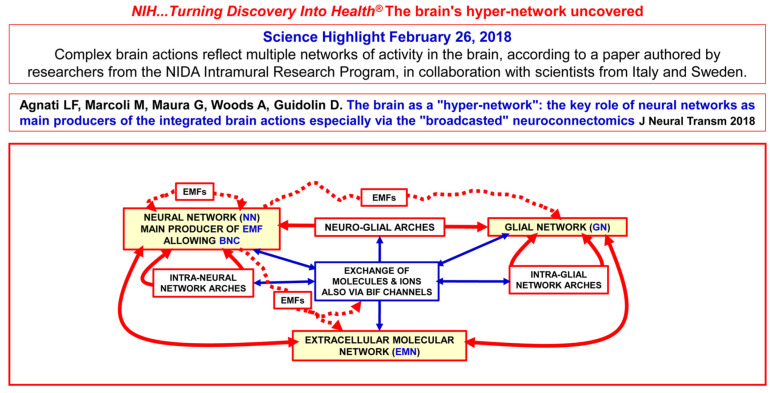
Schematic representation of the brain as a “hyper-network” formed by the integrated assembly of neural, glial and extracellular molecular networks; these components are often organized in compartments of different sizes, delimited by plastic boundaries [73]. Reprinted with permission from [73]. Copyright 2018, Springer Nature. The extracellular molecular network (EMN) is produced and dynamically modulated by both neurons and glial cells. In turn, the EMN plays a role in the formation and dynamic modulation of neuro-glial, intra-neural and intra-glial arches. Assemblies of components of the three networks form compartments (i.e., functional modules) delimited by plastic boundaries. Compartments contain circuits organized according to a ″Russian doll pattern″. Hence, macro-scale, meso-scale, micro-scale and nano-scale circuits can be described within each compartment. Highlighted by the National Institute of Health (NIH), Science Highlight and by the National Institute on Drug Abuse (NIDA). https://nida.nih.gov/news-events/science-highlight/brains-hyper-network-uncovered, accessed on 26 February 2018. BIF: brain interstitial fluid; BNC: broadcasted neuro-connectomics; EMFs: electromagnetic fields.

The morpho-functional data yielded by the above-mentioned multi-faceted approaches have also opened up new epistemological horizons. In particular, the conceptual model of the brain as a hyper-network (BHN, see [73]) has been proposed; this is schematically illustrated in Figure 2, and some of its main features are briefly discussed below.

**Figure 3:**
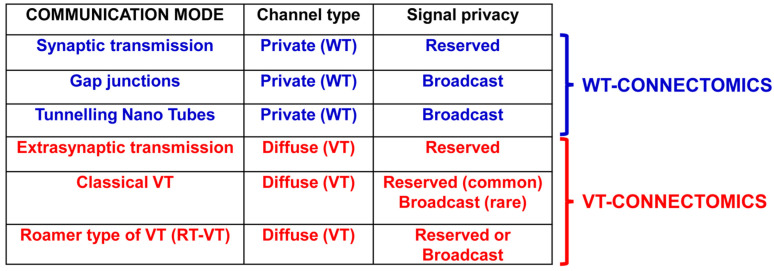
Main characteristics of the communication modes in cellular networks. Different types of wiring transmission and volume transmission modes.

As mentioned above, the main characteristics of the modes of communication between nodes in cellular networks are crucial to describe connectomics [82,83]. Our group has proposed a preliminary classification of the modes of communication between nodes in cellular networks, as reported in Figure 3.

*Channel type:*

- Private channel: physically delimited pathway between two nodes of the network
- Diffuse channel: the whole available space between the network nodes is potentially used to exchange signals

*Signal privacy:*

- Reserved signal: Signal needing a specific “decoder” in order to be decrypted. Neurotransmitters and, more generally, signals using specific receptor systems are of this type
- Broadcast signal: “Public” signal, i.e., interpreted by all the elements that it can reach. Physical processes (e.g., pressure waves) or membrane permeable molecules (e.g., oxygen) are of this type

Note that the roamer type of VT via the migration of extra-cellular vesicles and their internalization into target cells can transfer recognition/decoding apparatuses. Thus, the roamer type of VT can cause transient acquisition by the target cell of a new phenotype-like neurochemical fingerprint.

VT: volume transmission; WT: wiring transmission.

An aspect of basic theoretical and practical importance concerns the possible conceptual similarities and differences between the brain and artificial computing devices [84,85,86]. This issue will be discussed further in the concluding part of the present paper. Indeed, two questions have been examined: whether it is possible to map out brain regions specialized in carrying out some specific tasks; and whether the integrative functions of the brain are conceptually different from those of artificial intelligence devices, which in many instances can carry out tasks that only human beings were once considered able to perform (see [87,88,89,90]). This issue is of basic importance, not only because of its practical implications, but also with regard to investigations on possible future interplays between humans and artificial intelligence (e.g., [90]).

**Figure 4:**
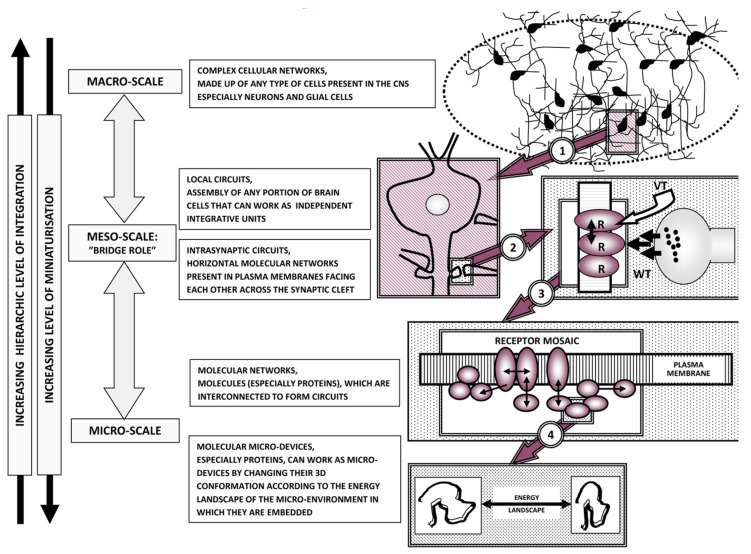
Schematic representation of miniaturization and hierarchic criteria. Three main miniaturization levels, i.e., the macro-, meso- and micro-scales, are illustrated. Modified from [14].

Concerning the morpho-functional organization of the brain, a recent paper [73] proposed the heuristic view that brain functions result from an integration of the information handling of the above-mentioned networks at different levels of miniaturization. Indeed (see Figure 4), brain structure appears to display a hierarchical, nested, “Russian doll” architecture [14,73,91]:

− Macro-scale: brain areas and, in greater detail, functional modules (see [73] for a definition of functional modules)
− Meso-scale: local circuits formed by the assembly of portions of brain cells, which can work as independent integrative units; a special role is played by synaptic clusters
− Micro-scale: in particular, penta-partite synapses formed by pre- and post-synaptic membranes, extracellular molecules and astrocytic processes
− Nano-scale: mainly protein-–protein allosteric interactions, as occur in receptor mosaics (RMs—proposed by Agnati and Fuxe and collaborators in the 1980s [92]; formal RM models were then suggested [93]—that often operate as crucial nodes in complex cellular networks.

**Figure 5:**
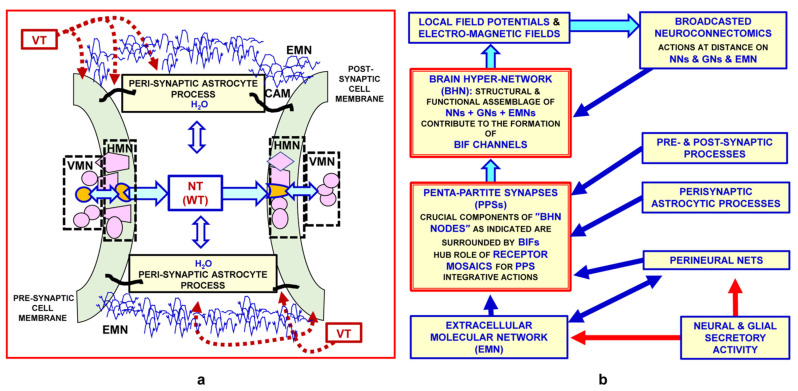
The penta-partite synapses. (**a**) Schematic representation of the main morpho-functional features of the penta-partite synapse. As schematically illustrated in the figure, astrocytes, extracellular matrix and neurons make up penta-partite synapses. Their integrative actions are also modulated by VT signals reaching the synaptic contacts via the extracellular fluid channels that impinge on them. The synaptic cleft and the classical pre- and post-synaptic sides of the synapse are of basic importance in the integrative function of the synaptic contacts. The peri-synaptic astrocytic processes and the specific cell-adhesion molecules that accumulate at pre- and post-synaptic sites also affect synaptic signalling, as do the microglial processes (not shown in the scheme). See text for further details and discussion. Modified from [73]. Adapted with permission from [73]. Copyright 2018, Springer Nature. (**b**) Glial cells, extracellular matrix and neurons make up the penta-partite synapses (PPSs). The scheme illustrates the heuristic hypothesis that PPSs are crucial components of the brain hyper-network nodes and are surrounded by brain interstitial fluid channels. See text for further details and a discussion of the most important implications of the present hypothesis. Modified from [73]. Adapted with permission from [73]. Copyright 2018, Springer Nature. BHN: brain hyper-network; BIF: brain interstitial fluid; CAM: cell-adhesion molecule; EMFs: electromagnetic fields; EMN: extracellular molecular network; HMN: horizontal molecular networks; GNs: glial networks; LFPs: local field potentials; NNs: neural networks; NTs: neurotransmitters; VMNs: vertical molecular networks; VT: volume transmission; WT: wiring transmission.

As mentioned above (see also Figure 5a,b), the penta-partite synapses are crucial components of the BHN nodes, and are endowed with extraordinary plasticity.

It has been demonstrated both theoretically and experimentally that the synapse can modulate its integrative actions in different ways [94]:

- Electrical signals from the pre-synaptic side can affect the post-synaptic side by means of induction;
- Electrical signals can be conducted by the extracellular fluid (electrotonic currents);
- A chemical mediator (neurotransmitter) can cross the synaptic cleft;
- Transient connection can take place between the pre-synaptic and post-synaptic neuron and also via the extracellular matrix surrounding the synaptic contact; the matrix is part of the extracellular molecular network, and affects pre- and post-synaptic morpho-functional aspects of some synaptic contacts [94].

Against this background, the proposed BHN prompted experimental studies (in which our group was significantly involved) of its morpho-functional organization also at the nano-scale level. This last issue will be summarized in the section that follows. Finally, some speculative heuristic hypotheses, based mainly on these data and their epistemological limits, will be briefly discussed.

## 3. Experimental Contributions to Investigations of the Morpho-Functional Organization of Brain Networks at Different Levels of Miniaturization

In recent decades, significant insights into brain architecture have stemmed mainly from two experimental approaches:

− Visualization of brain structures at different levels of miniaturization—from the cell networks to the molecular levels—by means of chemical neuroanatomical methods;
− Computer-assisted image analysis of the structures visualized.

Experimental evidence yielded by the synergic interactions of these approaches led not only to representations of the organization and function of the brain network at different levels of miniaturization, but also to the development of inclusive models of the brain’s integrative functions [73].

In other words, the basic task is to ascertain how far computer-assisted analyses of the morphology of brain structures can enable us to surmise their functional role in the network and in some specific integrative functions of the brain.

In particular, as mentioned above (see Figure 3), this research has led to the proposal of a broader view of communication in the brain, involving, in addition to synaptic transmission, a diffuse mode of inter-cellular communication (so-called volume transmission, VT) that, in some way, may remind us of Golgi’s proposed integrative function of a “diffuse nerve network” [22]. Uptake mechanisms function as important ways of limiting the diffusion of a neurotransmitter outside the synapse and its ability to reach distant targets through VT [95]. The monoamines dopamine, noradrenaline and serotonin operate mainly via VT in the mammalian brain; the importance of their uptake mechanisms is indicated, e.g., by the finding that monoamine uptake blockers increase the synaptic levels of these monoamines and function as antidepressants (this appears to be especially true of serotonin and noradrenaline) and by the fact that some drugs of abuse target dopamine transporters (see [96]). In the case of amino acid transmitters (e.g., glutamate and GABA), both neuronal and glial uptake play crucial roles in maintaining functional brain connectomics. For example, astrocytic uptake mechanisms control extracellular glutamate levels at synapses and prevent excessive glutamate receptor activation and excitotoxicity [97]. Motility of the peri-synaptic astrocytic processes—in response to synaptic neuronal activity [98]—and the regulation of synaptic permeability is a further mechanism by which astrocytes can control the synapse, making signaling regulation by astrocytes in brain circuits even more complex.

Thus, the next section will provide a historical and introductory presentation of modes of communication in the brain, together with some of the experimental evidence obtained on this topic.

### 3.1. The “Mismatch” in Several Histochemical Images between the Nerve Terminals, and Hence the Neurotransmitter Stores, and Their Respective Decoding Receptors: A Basic Datum in the Proposal of Non-Synaptic Transmission, i.e., Volume Transmission

Volume transmission (VT) was first hypothesized following the observation of transmitter-receptor mismatches, mainly in double-immuno-labelling experiments. The first evidence of VT was the lack of correlation between the distribution of enkephalin and beta-endorphin immunoreactive nerve terminals and the distribution of opiate receptors [99]. As already stressed in the first papers on VT, mismatch is a necessary, but not sufficient, condition to ascertain VT [100]. Some crucial aspects, such as the existence of sources of VT signals, of preferential pathways in the CNS and of energy gradients that enable the VT signal to migrate along extracellular fluid pathways, have therefore been investigated.

Against this background, as early as the 1980s, a dichotomous classification of inter-cellular communication modes was introduced, namely ‘wiring transmission’ (WT) and “VT” (see Figure 3 for the main aspects). In brief, it was proposed that WT was characterized by a well-identified physical channel (i.e., “a wire”) connecting the cell source of the signal to the target cell, while VT was characterized by the possible three-dimensional diffusion of electro-tonic signals (as Golgi proposed, based on Volta’s studies of second-class electrical conductors), of transmitters, trophic factors, ion signals and gases, which reached the brain and/or were released in the extracellular space and cerebrospinal fluid by different types of cells, and diffused in many instances in preferential fluid channels (see below).

In view of the experimental contributions of our group to this subject, let us briefly mention some main features of VT communication in the CNS [91,101,102].

#### 3.1.1. Types of VT Signals

Chemical signals: Neurotransmitters, neuromodulators, growth factors, hormones, ions (e.g., Ca^2+^ ions) and gases (e.g., NO, CO_2_, CO). Obviously, since the basic process in this communication mode is diffusion in the medium, it is important to distinguish lipophilic from hydrophilic VT signals. Moreover, the former can also diffuse through cell membranes (i.e., they have a large space of diffusion), while the latter are largely confined [103,104,105].

Physical signals: Pressure waves (see data of [106]) and temperature waves—since local perturbations in the metabolic activity of brain cells can cause temperature waves between activated and surrounding brain tissue [107] and electrotonic currents (local field potentials [108,109]). Accordingly, it has been demonstrated that spike codes and non-spike codes coexist. Specifically, field potentials are sometimes also signals for neighboring cells, since they modulate the electrical and chemical properties of the plasma membrane of neighboring cells [108,109]. The propagation of an action potential causes a depletion of charge in the intracellular space and a net acquisition of charge in the extracellular space, thereby generating a difference in potential across the membrane and, consequently, axial current flow from each axonal segment to the following one. Thus, an inhomogeneous and time-varying electromagnetic field is generated around the neurons. Recent studies aimed at modeling this process (see [110]) indicate that these fields can reach strengths of 3.0 × 10^−12^ T at the nodes of Ranvier and of about 2.5 × 10^−12^ T at the myelinated segments, which are homogeneous up to a distance of several microns from the cell membrane and are able to modulate cell-to-cell communication.

As shown in Figure 3, a peculiar mode of intercellular communication is the so-called roamer type of VT [74,111,112]. The limits of neural connectomics (which will be discussed below) are further emphasized by the fact that exosomes can allow the intercellular transfer of elements of the recognition/decoding apparatus of cells (e.g., receptors), leading to a transient phenotypic change in the target cell [74,111,113].

#### 3.1.2. Pathways of VT-Signal Migration

Isotropic diffusion in the extracellular space of the brain. This usually results from a process of diffusion in a fairly homogeneous medium.

Preferential pathways in the extracellular space of the brain. Anisotropic migration occurs mainly along nerve bundles, especially along white matter tracts and in the perivascular spaces.

Cerebrospinal fluid as a “vector” conveying VT signals. Indeed, both solutes and solvents diffuse from the cerebrospinal fluid to the brain interstitial fluid, crossing both the pial and ventricular ependyma. Aquaporin 4 (AQP-4) plays a particular role, since these water-channel proteins are selective pores controlled by peptidergic signals such as atrial natriuretic peptide and vasopressin [114,115].

It should be considered that barriers exist to control VT-signal migration from the cerebrospinal fluid to the brain interstitial fluid.

#### 3.1.3. Energy Gradients for VT-Signal Migration

Concentration Gradients (ref. [104] and References Therein)
Gradients of Electrical Potentials (for Charged Signals) (ref. [104] and References Therein)
Pressure Gradients (ref. [104] and References Therein)
Temperature Gradients (ref. [104]; See also [107])

With regard to energy gradients, it is important to distinguish macro-gradients (e.g., between brain areas) from “micro-gradients” (e.g., between a synapse and the surrounding environment, especially in synaptic clusters).

#### 3.1.4. Decoding Systems for VT-Signals

Private decoding systems: receptors, enzymes, ion channels, temperature-sensitive receptors, pressure-sensitive receptors (stretch-sensitive ion channels, e.g., magnocellular neurons of the hypothalamus that respond to blood osmolarity and hence to alterations in the volume of neurons and stretching of their plasma membrane [116]).

Non-private decoding systems: membrane polarization, chemical reactions (affected by temperature alterations with a Q10 value of about 2.3 [107]).

Thus, VT often takes place without dedicated communication channels, hence without space-filling requirements, and often uses energy gradients that are also used for other purposes, e.g., for renewal of the extracellular fluid. Indeed, the generation of physical VT signals (e.g., pressure waves in cerebral blood vessels and temperature waves) and the migration of chemical VT signals may, in many respects, be the by-products of phenomena occurring in the brain to fulfill different tasks. In other words, a “tinkering” process [117] occurs in the brain, allowing a mode of intercellular communication that is both energetically and spatially highly efficient.

In sum, WT is usually more “costly” than VT in terms of both space filling and energy consumption. Let us now examine the most prominent case of WT: the chemical synapse (e.g., [118]). In general, space filling consists of a dedicated channel, which usually involves an axon, a pre-synaptic nerve terminal and post-synaptic specialization. The energy needed is that required for the action potential, the synthesis and release of the transmitter and the decoding of this signal at the post-synaptic level. Indeed, since the pioneering work of McCulloch and Pitts [119], the chemical anatomy of neural networks, and particularly the organization of synaptic contacts, have been central to designing devices that are hypothetically capable of mimicking human cognitive capabilities; hence, it has been of basic importance to the foundation of artificial intelligence technologies [120]. However, as already briefly mentioned, the synaptic contacts do not comply with Dale’s principle; rather, they are highly complex morpho-functional structures capable of dynamic changes and endowed with multiple transmission lines that can be modulated by the above-mentioned biochemical and physical signals, which act on neurons, glial cells and the extracellular matrix (e.g., [73]).

An important modulatory role in intercellular communication in the central nervous system is played by the so-called horizontal molecular networks at the plasma membrane level. These will be the focus of the next section. In this context, direct RRIs constitute significant “plastic components” of these integrative mechanisms, and some experimental evidence of their importance, together with the conceptual implications of this view, will be briefly discussed.

### 3.2. Evidence of the Existence of Horizontal Molecular Networks at Cell Membrane Levels and of the Integrative Role of GPCR Aggregates

**Figure 6:**
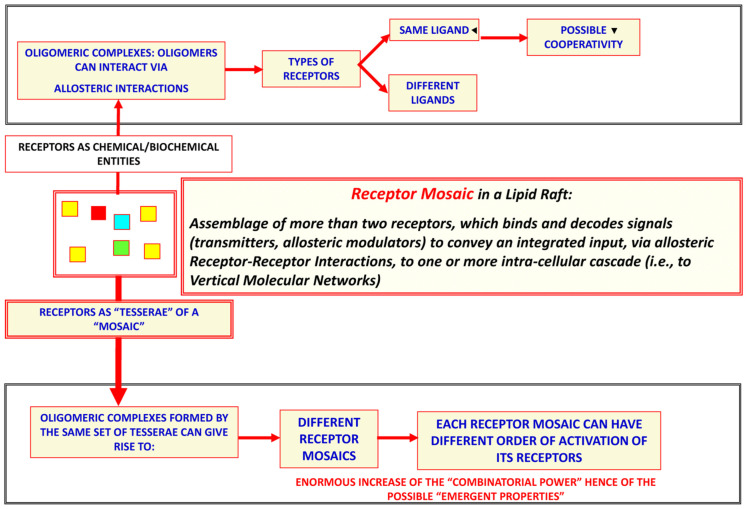
CGPRs as monomers, dimers or receptor mosaics. Chemical and biochemical aspects: homomers vs. heteromers and cooperativity vs. non-cooperativity. The integrative actions of a receptor mosaic depend not only on its stoichiometry but also on the spatial organization (topology) and order of activation of its tesserae. Furthermore, the roamer type of volume transmission can cause a transient acquisition by the target cell of a new phenotype-like plasticity.

It should be underlined that, as pointed out above, not only a neuron terminal can release different signals, according to some not yet well clarified mechanisms at the pre-synaptic level; there are also different decoding mechanisms at the post-synaptic level, as will be discussed below. Thus, in the 1980s [92,93,94,95,96,97,98,99,100,101,102,103,104,105,106,107,108,109,110,111,112,113,114,115,116,117,118,119,120,121,122,123], our group proposed that at both the pre-synaptic and post-synaptic levels, micro-domains could operate as “intelligent interfaces” to mediate interactions between the extra-cellular and the intra-cellular environments. In this respect, together with other groups (e.g., [16,124,125] and references cited therein), we demonstrated that a specific role was played by GPCRs in the horizontal molecular networks present in these membrane micro-domains. Indeed, GPCRs can be inserted into the cell plasma membrane as monomers, dimers or oligomers, and these different molecular arrangements have important functional implications (Figure 6).

As mentioned above, our research focused on the horizontal molecular networks characterized of GPCR aggregates, which can operate as an integrative unit via allosteric RRI (for articles on this subject, see [19,124]). These receptor complexes provide an “intelligent” interface with integrative functions, resulting in the modulation of the vertical molecular networks transducing the incoming signal, and, as a consequence, the cell response. Thus, a new field has been proposed for the integration of extracellular signals at the plasma membrane level (for a discussion of the topic see, e.g., [16,18]).

It should be noted that key preliminary experimental evidence for RRIs is the demonstration that two receptors involved in the RRI process are in close proximity (<10 nm); this close co-localization allows a possible allosteric interaction between the molecules of the two receptors after the ligand binds to one receptor. However, the early evidence of RRIs was indirect, being based on coarse co-localization revealed by computer-assisted image analyses of double-stained immunocytochemical preparations. The hypothesis of RRIs, however, was supported by biochemical data, which demonstrated that, in membrane preparations from discrete brain regions, the binding of a ligand to one receptor could modulate the binding characteristics of the other receptor [126,127]. Functional studies carried out in vivo in physiological and pathological animal models further supported this evidence, highlighting the functional relevance of the in vitro findings [128,129].

In the last few decades, new biophysical techniques have been developed in order to detect the spatial proximity of protein molecules (<10 nm), such as: energy transfer-based methods, bimolecular luminescence or fluorescence complementation, fluorescence correlation spectroscopy, total internal reflection fluorescence microscopy, co-immunoprecipitation or assays based on bivalent ligands and in situ proximity ligation assays (ref. [70] and bibliography cited therein). These techniques have provided direct experimental support for the RRI hypothesis and for the existence of receptor complexes at the cell membrane.

Here, we must consider some peculiar aspects of RRIs that lead to GPCR oligomerization at both the neural and astrocyte plasma membrane, as reported mainly by our group.

The basic phenomenon underlying RRI is allostery, which can occur in multimeric proteins. As pointed out by Monod, “allostery” for protein functions should be considered “the second secret of life”, since it is second in importance only to the genetic code [130,131]. A possible consequence of allostery is cooperativity, which occurs when the binding of a ligand to a receptor alters the conformational characteristics, and hence the affinity, of another receptor of the RM (see, e.g., [132]). It should be noted, as discussed in previous papers (see, e.g., [124]), that it is also possible to have receptor monomers, receptor co-localization without heteromerization, or receptor heteromerization without allosteric interactions, when an interaction with a receptor does not lead to conformational changes in other receptors of the complex [133]. However, when allosteric interactions occur in an RM, an integrative nanoscale center operates at the cell plasma membrane level (for a discussion of the topic see, e.g., [16,18,70,91,124,134,135,136]).

Owing to allosteric interactions, proteins embedded in and/or associated with the cell plasma membranes can become organized in horizontal molecular networks, which can also operate as autonomous integrative modules capable of performing specialized tasks according to the specific topologies in which the proteins involved form a “mosaic” [135]. Such mosaics may last for longer or shorter periods of time, being plastic assemblies that can also undergo reshuffling, including the addition of new proteins (“tesserae” of the mosaic) or alteration of their topology (for a discussion of the topic see, e.g., [16,18,124]).

It has been demonstrated that GPCR oligomerization can already affect the functional features of the GPCR monomers at the level of dimers. A paradigmatic case is that of the opioid receptor subtypes, which acquire new biochemical and functional characteristics when they form heterodimers. Indeed, k − δ and δ − μ opioid receptor heteromers constitute a new receptor endowed with different characteristics, in terms of ligand binding and functional properties (e.g., G-protein coupling), from those of the contributing monomers [137,138]. The same phenomenon has been reported for the D1R-D2R heteromer, with a shift from Gs (D1R) and Gi/o (D2R) to Gq/11 coupling [139]. These emergent properties enhance the RMs in comparison with component monomers; in particular, the following ones properties should be mentioned (Figure 6):

- The possible appearance of new binding sites or binding characteristics in each monomer (refs. [121,123,126,127]; see also [140]);
- Different localization of the RM at plasma membrane levels, in comparison with the isolated monomers (e.g., preferential localization in the lipid rafts) [141,142];
- Different turnover rate and desensitization of the monomers in the RM in comparison with the isolated GPCRs [143,144,145];
- The possible existence of a “Hub Receptor” in an RM that is made up of three or more GPCRs. The Hub Receptor has been defined as the GPCR that can interact with multiple molecules, including receptors of the RM or membrane-associated proteins [18,144,145,146,147,148,149].

It has been demonstrated that RRIs can modulate neuron–neuron, glia–neuron and glia–glia intercellular communication, with significant effects on the synaptic activity and integrative functions of the brain networks. Investigations in this field may be of great importance, owing to the likely implications for new pharmacological approaches, especially in view of the role of the glia in maintaining the integrity of neural networks [70].

Here, we have focused on GPCRs, as they are the largest family of signaling receptors in eukaryotes and are the targets for about 35% of approved drugs [150,151,152]. However, we are well aware that signal integration is complicated by the interactions of membrane GPCRs and ionotropic receptors [153]. For example, ionotropic and metabotropic glutamate receptors are co-expressed at individual synapses and work in concert, enabling precise temporal modulation of post-synaptic excitability and plasticity. Both receptors have emerged as potential drug targets in the treatment of brain disorders, including schizophrenia, depression and addiction, allowing a more holistic understanding of neural glutamate signaling [154]. To understand the differential contribution of these receptors to synaptic transmission, we need to consider, in addition to their signaling properties, the mechanisms controlling spatial segregation of the receptor types within synapses; these mechanisms are only beginning to be explored in the context of synapse organization [155].

Insight into the integrative functioning of the brain, from the level of allosteric RRIs to the hierarchical architecture of the brain, may help us to understand both the functioning of the healthy brain and its dysfunction in pathological conditions. Indeed, receptor complexes are highly dynamic assemblies. In the healthy brain, it has been suggested that the reorganization of receptor complexes at the post-synaptic level is the basis for learning and memory, and that long-term memory may be linked to the consolidation of these complexes into long-lived complexes with conserved allosteric RRIs (see [156]). Below, we report some examples of heteroreceptor complex dysfunction in pathological conditions, and therefore, of how the concepts proposed here could be applied to neuropathology and therapeutics in human brain diseases. It has been claimed that the development of major depression involves an imbalance of 5-HT1 receptor activity and of receptor complexes containing the 5-HT1A receptor [156]. Another example concerns schizophrenia [156]; indeed, a reduced density of A2A-D2 heteromers in the post-mortem caudate nucleus of patients with schizophrenia has been reported [157]. Moreover, different anti-psychotic drugs affect A2A-D2 heteromerization differently, changing the density and stability of GPCR heteromers, which are therapeutic targets in psychosis [158]. The finding of increased A2A-D2 heteromerization in the post-mortem caudate of Parkinson’s disease patients might help us to better understand the etiology of the disease and to design selective pharmacotherapeutic strategies [159]. Indeed, the action of drugs on GPCR complexes at penta-partite synapses—i.e., allosteric intervention on GPCRs—can increase the selectivity of pharmacological treatments (see [160]). Apart from reducing adverse side effects [161], allosteric ligands can provide greater receptor subtype selectivity and temporal selectivity, and are attractive targets for drug development [13].

Furthermore, it should be borne in mind that reactive astrocytosis and maladaptive changes of components of the penta-partite synapse occur in the pathological process of various neurodegenerative diseases, hence, an investigation of the role of reactive astrocytes and their alteration in various neurodegenerative states may shed light on the prevention and treatment of these diseases [68,161,162].

Thus, Complex Cellular Networks should be considered in the context of their integrative mechanisms for the reciprocal release and decoding of multiple signals at the different miniaturization levels [16,146].

Brain-wide connectivity, as pointed out above, can be described at the macroscopic, mesoscopic and microscopic levels, and therefore, the neuro-connectomics approach has been proposed for this purpose. Specifically, Sporns underlines that “*Connectome maps explicitly aim at representing the brain as a complex network, a collection of nodes and their interconnecting edges*”, and mentions some main aspects, such as the temporal dynamics of functional brain connectivity, as the criteria for defining brain areas that also transiently play a role in the execution of a certain brain function, and the hierarchical organization of the brain networks. Sporns also considers how the different miniaturization levels are integrated [82]. It should be pointed out that the task of connectomics is very arduous, since it should integrate incoming information from the internal and external environments with memory stores and the surmising of future scenarios. However, as underlined by Lord and co-authors [83] “*In order to promote survival, the brain must be capable of integrating a wide range of incoming stimuli from its environment and seamlessly ‘bind’ this complex stream of information into meaningful internal representations that are then used to plan for the next action*.” It should be mentioned that Denis Diderot wrote almost the same in his book “*Le rêve de d’Alembert”* (ref. [163] publication date: 1830).

Quantitative neuroanatomy does not try to solve these problems; it merely underlines the complexity of the field and the new openings that may emerge from the synergic impact of computer-assisted analyses of the chemical neuroanatomy images produced by recently developed instruments. Indeed, detailed images of the different levels of miniaturization of brain networks are available, and their analysis by means of the recent artificial intelligence approaches should yield a better understanding of the brain’s integrative functions.

## 4. Future Investigations on Integrative Functions of the Brain

In sum, it has been suggested that the integrative functions of the CNS are the result of the complex dynamics of complex cellular networks in which neurons and other resident cells interact through different types of channels that convey chemical and physical signals, and that information can be processed at different hierarchical levels, i.e., at the level of interconnected brain areas, of local cellular networks, and also at molecular levels. This was the basis on which a previous paper proposed the concept of the “brain as a hyper-network”, whereby neural networks probably play a key role as principal producers of the brain’s integrated actions, especially via “broadcasted” neuro-connectomics [73].

However, brain connectomics is a plastic set of edges and nodes that are also structurally and functionally modulated by signals released by peripheral organs and which continuously impinge on the brain. Indeed, experimental evidence has shown that peripheral organs, such as the heart (especially via vagal afferences and arterial pulses) and the gut (especially via the action of the microbiota), can also modulate brain functions. These profound effects on brain connectomics are also under investigation with regard to their pathological implications (see, e.g., [164,165,166] and bibliography cited therein). The microbiota–gut–brain axis, in particular, has been seen to play a role in the pathophysiology of numerous mental and neurological diseases [167]. In support of this view, it has been shown that the diversity of the microbiota is reduced in aging-related diseases such as depression, Alzheimer’s disease, Parkinson’s disease and multiple sclerosis [168,169].

## 5. Final Comment: Epistemological Considerations

As discussed in the premises of the present paper, Sherrington’s hypothesis of synaptic contacts prompted McCulloch and Pitts [119] to propose the application of a Boolean model to the integrative functions of neural networks and to speculate on the formation of memory engrams. In this context, Hebb proposed his theory of memory, which was subsequently largely mentioned (see, e.g., [170] and bibliography cited). However, while subsequent experimental data on the brain’s morpho-functional organization did not completely reject the Boolean model of the integrative functions of neural networks, they clearly demonstrated its inadequacy. Furthermore, as reported in the text, illustrated in Figure 2, Figure 5a,b and Figure 6, and mentioned in the bibliography, the available experimental data have revealed that the brain’s morpho-functional organization is extremely complex and variegated. This evidence raises a basic epistemological question, since not only neuronal networks but also different interacting cellular and molecular networks operate in the brain at several levels of miniaturization, and several different chemical and physical signals enable the brain’s integrative actions via their multiple cellular and molecular mechanisms. Specifically, engram formation probably takes place via different forms of interaction and plasticity of these cellular and molecular networks, and hence through the cooperation of multiple agents at different miniaturization levels.

Indeed, it should be underlined that brain plasticity is multi-faceted; in addition to the well-characterized synaptic plasticity, we can also observe glial cell plasticity, extracellular fluid channel plasticity, protein–protein allostery and, as shown by our group, a “phenotypic plasticity” obtained via the roamer type of VT (Figure 1 and Figure 6). Thus, with regard to the brain’s integrative actions, we have introduced several heuristic models, in particular, Turing’s unorganized machine (see Figure 1), which can operate at each miniaturization level, and hence also at the molecular level, to control the allosteric interactions in RMs [16,136,171,172]. Furthermore, the astonishingly complex and multi-faceted processes through which the brain performs computations and builds a model of the environment are the subject of hypothesis and investigation. Of the greatest importance are:

− The Penrose and Hameroff hypothesis that brain computations are basically due to quantum computations that involve hydrophobic areas of microtubules, whose electron clouds undergo orchestrated superposition and reduction, producing proto-conscious elements that become orchestrated into conscious experiences. The main aspects of this interesting hypothesis have recently been discussed by Schiffer [173];
− Friston’s free-energy principle, according to which a basic feature of any organism—from the single cell to the human brain—is that sensory inputs and memory stores are used to build a reliable model of the organism’s environment. Thus, Friston’s free-energy principle states that every living being, at every scale of organization, is driven by an imperative: to sample the world and to ensure that its predictions become a self-fulfilling prophecy. Friston argues that this imperative can be reduced to a mathematical function [174].

**Figure 7:**
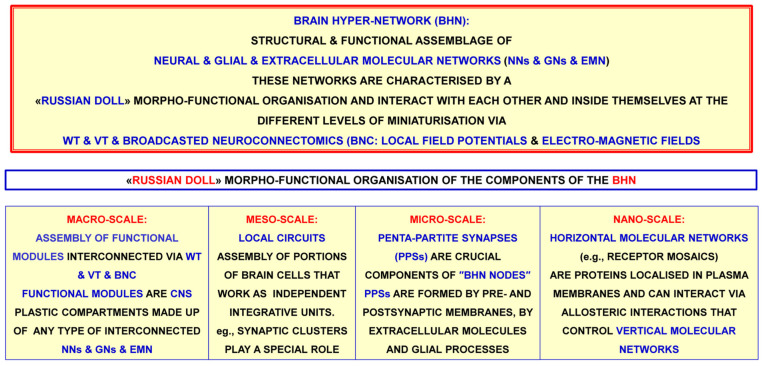
From the brain hyper-network to the brain’s integrative actions. A basic epistemological question regarding brain integrative actions: from the experimental evidence, is it possible to construct a complete formal model of the brain hyper-network (BHN) in order to investigate brain integrative actions? EMN: extracellular molecular network; HMNs: horizontal molecular networks; GNs: glial networks; NNs: neural networks; PPSs: penta-partite synapses; VT: volume transmission; WT: wiring transmission.

Against this astonishingly complex background of brain organization and function, what clearly emerges is a picture of the brain as a very peculiar system, in which genuine computational processes act in concert with non-computational dynamic processes, leading to continuous self-organization and remodeling under the action of external stimuli from the environment and from the rest of the organism (ref. [88]; see also Figure 7).

Investigation of this subject is highly demanding, but certainly rewarding on account of both the possible therapeutic implications and the intellectual satisfaction that scientific investigations can yield [175].

Thus, as a concluding reflection, we can mention Emily Dickinson’s great verse [176]:

  *The Brain—is wider than the Sky—*
  *For—put them side by side—*
  *The one the other will contain*
  *With ease—and You—beside—.*

## Data Availability

Not applicable.

## List of Abbreviations

BHN	Brain Hyper-Network
CNS	Central Nervous System
GPCR	G Protein-Coupled Receptor
RM	Receptor Mosaic
RRI	Receptor–receptor interaction
VT	Volume Transmission
WT	Wiring Transmission

## References

[1] Kuhn T.S. (1962). The Structure of Scientific Revolutions.

[2] Kuhn T.S. (1996). The Structure of Scientific Revolutions.

[3] Björklund A., Hökfelt T. (1983). Handbook of Chemical Neuroanatomy, Vol. 1: Methods in Chemical Neuroanatomy.

[4] Kuhar M.J., Unnerstall J.R., De Souza E.B. (1985). Receptor mapping in neuropharmacology by autoradiography: Some technical problems. NIDA Res. Monogr..

[5] Agnati L.F., Fuxe K. (1985). Quantitative Neuroanatomy in Transmitter Research.

[6] Sporns O. (2012). Discovering the Human Connectome.

[7] Krishnan V., Nestler E.J. (2008). The molecular neurobiology of depression. Nature.

[8] Finnerup N.B., Kuner R., Jensen T.S. (2021). Neuropathic pain: From mechanisms to treatment. Physiol. Rev..

[9] Yang M., Yang Z., Wang P., Sun Z. (2021). Current application and future directions of photobiomodulation in central nervous diseases. Neural Regen. Res..

[10] May L.T., Leach K., Sexton P.M., Christopoulos A. (2007). Allosteric modulation of G protein-coupled receptors. Annu. Rev. Pharmacol. Toxicol..

[11] Hauser A.S., Attwood M.M., Rask-Andersen M., Schiöth H.B., Gloriam D.E. (2017). Trends in GPCR drug discovery: New agents, targets and indications. Nat. Rev. Drug Discov..

[12] Nussinov R., Tsai C.J. (2013). Allostery in disease and in drug discovery. Cell.

[13] Nickols H.H., Conn P.J. (2014). Development of allosteric modulators of GPCRs for treatment of CNS disorders. Neurobiol. Dis..

[14] Agnati L.F., Baluška F., Barlow P.W., Guidolin D. (2009). Mosaic, self-similarity logic and biological attraction principles: Three explanatory instruments in biology. Commun. Integr. Biol..

[15] Milligan G. (2009). G protein-coupled receptor hetero-dimerization: Contribution to pharmacology and function. Br. J. Pharmacol..

[16] Agnati L.F., Guidolin D., Leo G., Carone C., Genedani S., Fuxe K. (2010). Receptor-receptor interactions: A novel concept in brain integration. Prog. Neurobiol..

[17] Lane J.R., Donthamsetti P., Shonberg J., Draper-Joyce C.J., Dentry S., Michino M., Shi L., López L., Scammells P.J., Capuano B. (2014). A new mechanism of allostery in a G protein-coupled receptor dimer. Nat. Chem. Biol..

[18] Farran B. (2017). An update on the physiological and therapeutic relevance of GPCR oligomers. Pharmacol. Res..

[19] Guidolin D., Marcoli M., Tortorella C., Maura G., Agnati L.F. (2018). G protein-coupled receptor-receptor interactions give integrative dynamics to intercellular communication. Rev. Neurosci..

[20] Guidolin D., Marcoli M., Tortorella C., Maura G., Agnati L.F. (2019). Receptor-Receptor Interactions as a Widespread Phenomenon: Novel Targets for Drug Development?. Front. Endocrinol..

[21] Slosky L.M., Caron M.G., Barak L.S. (2021). Biased Allosteric Modulators: New Frontiers in GPCR Drug Discovery. Trends Pharmacol. Sci..

[22] Agnati L.F., Genedani S., Leo G., Rivera A., Guidolin D., Fuxe K. (2007). One century of progress in neuroscience founded on Golgi and Cajal’s outstanding experimental and theoretical contributions. Brain Res. Rev..

[23] Mazzarello P. (2010). Golgi C: A Biography of the Founder of Modern Neuroscience.

[24] Golgi C. (1906). The Neuron Doctrine—Theory and Facts; Nobel Lecture. https://www.nobelprize.org/prizes/medicine/1906/golgi/lecture/.

[25] Cajal S.R. (1906). The Structure and Connexions of Neurons; Nobel Lecture. https://www.nobelprize.org/prizes/medicine/1906/cajal/lecture/.

[26] Sherrington C.S. (1906). The Integrative Action of the Nervous System.

[27] Zeng H., Sanes J.R. (2017). Neuronal cell-type classification: Challenges, opportunities and the path forward. Nat. Rev. Neurosci..

[28] Dale H.H. (1934). Chemical Transmission of the Effects of Nerve Impulses. BMJ.

[29] Dale H. (1935). Pharmacology and Nerve-Endings. Proc. R. Soc. Med..

[30] Eccles J.C. (1957). The Physiology of Nerve Cells.

[31] Strata P., Harvey R. (1999). Dale’s principle. Brain Res. Bull..

[32] Palay S.L., Wissig S.L. (1953). Secretory granules and Nissl substance in fresh supraoptic neurones of the rabbit. Anat. Rec..

[33] Palade G.E., Palay S.L. (1954). Electron microscope observations of interneuronal and neuromuscular synapses. Anat. Rec..

[34] Palay S.L. (1956). Synapses in the central nervous system. J. Biophys. Biochem. Cytol..

[35] Falck B., Hillarp N.-Å., Thieme G., Torp A. (1962). Fluorescence of catechol amines and related compounds condensed with formaldehyde. J. Histochem. Cytochem..

[36] Dahlstrom A., Fuxe K. (1964). Localization of monoamines in the lower brain stem. Experientia.

[37] Anden N.E., Carlsson A., Dahlstroem A., Fuxe K., Hillrap N.A., Larsson K. (1964). Demonstration and mapping out of nigro-neostriatal dopamine neurons. Life Sci..

[38] Fuxe K. (1965). Evidence for the existence of monoamine neurons in the central nervous system: IV. Distribution of monoamine nerve terminals in the central nervous system. Acta Physiol. Scand..

[39] Fuxe K. (1965). Evidence for the existence of monoamine neurons in the central nervous system: 3. The monoamine nerve terminal. Z. Zellforsch. Mikrosk. Anat..

[40] Dahlström A., Fuxe K. (1965). Evidence for the existence of monoamine neurons in the central nervous system: II. Experimentally induced changes in the intraneuronal amine levels of bulbospinal neuron systems. Acta Physiol. Scand. Suppl..

[41] Benfenati F., Cimino M., Agnati L.F., Fuxe K. (1986). Quantitative autoradiography of central neurotransmitter receptors: Methodological and statistical aspects with special reference to computer-assisted image analysis. Acta Physiol. Scand..

[42] Agnati L.F., Fuxe K., Benfenati F., Zini I., Zoli M., Fabbri L., Härfstrand A. (1984). Computer assisted morphometry and microdensitometry of transmitter identified neurons with special reference to the mesostriatal dopamine pathway—I. Methodological aspects. Acta Physiol. Scand. Suppl..

[43] Zoli M., Zini I., Agnati L.F., Guidolin D., Ferraguti F., Fuxe K. (1990). Aspects of neural plasticity in the central nervous system—I. Computer-assisted image analysis methods. Neurochem. Int..

[44] Pert C.B., Kuhar M.J., Snyder S.H. (1975). Autoradiograhic localization of the opiate receptor in rat brain. Life Sci.

[45] Hökfelt T., Johansson O., Goldstein M. (1984). Chemical anatomy of the brain. Science.

[46] Yelnik J., Bardinet E., Dormont D., Malandain G., Ourselin S., Tandé D., Karachi C., Ayache N., Cornu P., Agid Y. (2007). A three-dimensional, histological and deformable atlas of human basal ganglia. I. Atlas construction based on immunohistochemical and MRI data. NeuroImage.

[47] Hökfelt T., Elfvin L.G., Elde R., Schultzberg M., Goldstein M., Luft R. (1977). Occurrence of somatostatin-like immunoreactivity in some peripheral sympathetic noradrenergic neurons. Proc. Natl. Acad. Sci. USA.

[48] Hökfelt T., Holets V.R., Staines W., Meister B., Melander T., Schalling M., Schultzberg M., Freedman J., Björklund H., Olson L. (1986). Coexistence of neuronal messengers—An overview. Prog. Brain Res..

[49] Furness J.B., Morris J.L., Gibbins I.L., Costa M. (1989). Chemical coding of neurons and plurichemical transmission. Annu. Rev. Pharmacol. Toxicol..

[50] Burnstock G. (2004). Cotransmission. Curr. Opin. Pharmacol..

[51] Svensson E., Apergis-Schoute J., Burnstock G., Nusbaum M.P., Parker D., Schiöth H.B. (2019). General Principles of Neuronal Co-transmission: Insights from Multiple Model Systems. Front. Neural Circuits.

[52] Hökfelt T. (1991). Neuropeptides in perspective: The last ten years. Neuron.

[53] Lundberg J.M. (1996). Pharmacology of cotransmission in the autonomic nervous system: Integrative aspects on amines, neuropeptides, adenosine triphosphate, amino acids and nitric oxide. Pharmacol. Rev..

[54] Hökfelt T., Broberger C., Xu Z.D., Sergeyev V., Ubink R., Diez M. (2000). Neuropeptides—An overview. Neuropharmacology.

[55] Cifuentes F., Morales M.A. (2021). Functional Implications of Neurotransmitter Segregation. Front. Neural Circuits.

[56] Kandel E.R. (1977). Cellular Basis of Behaviour.

[57] Kandel E., Koester J.D., Mack S.H., Siegelbaum S.A. (2021). Principles of Neuroscience.

[58] Agnati L.F., Fuxe K., Zoli M., Pich E.M., Benfenati F., Zini I., Goldstein M. (1986). Aspects on the information handling by the central nervous system: Focus on cotransmission in the aged rat brain. Prog. Brain Res..

[59] Kim S., Sabatini B.L. (2023). Analytical approaches to examine gamma-aminobutyric acid and glutamate vesicular co-packaging. Front. Synaptic Neurosci..

[60] Hnasko T.S., Edwards R.H. (2012). Neurotransmitter corelease: Mechanism and physiological role. Annu. Rev. Physiol..

[61] Tritsch N.X., Granger A.J., Sabatini B.L. (2016). Mechanisms and fuction of GABA co-release. Nat. Rev. Neurosci..

[62] Saunders A., Oldenburg I.A., Berezovskii V.K., Johnson C.A., Kingery N.D., Elliott H.L., Xie T., Gerfen C.R., Sabatini B.L. (2015). A direct GABAergic output from the basal ganglia to frontal cortex. Nature.

[63] Changeaux J.P., Christopoulos A. (2017). Allosteric modulation as a unifying mechanism for receptor function and regulation. Diabetes Obes. Metab..

[64] Virchow R., Virchow R. (1856). Ueber das granulirte Ansehen der Wandungen der Gehirnventrikel. Gesammelte Abhandlungen zur Wissenschaftlichen Medicin.

[65] Virchow R. (1858). Die Cellularpathologie in Ihrer Begründung auf Physiologische und Pathologische Gewebelehre.

[66] Lugaro E. (1907). Sulle funzioni della nevroglia. Riv. Pat. Nerv. Ment..

[67] Verkhratsky A., Nedergaard M. (2018). Physiology of astroglia. Physiol. Rev..

[68] Sofroniew M.V., Vinters H.V. (2010). Astrocytes: Biology and Pathology. Acta Neuropathol..

[69] Parpura V., Heneka M.T., Montana V., Oliet S.H.R., Schousboe A., Haydon P.G., Stout R.F., Spray D.C., Reichenbach A., Pannicke T. (2012). Glial cells in (patho)physiology. J. Neurochem..

[70] Guidolin D., Tortorella C., Marcoli M., Cervetto C., Maura G., Agnati L.F. (2021). Receptor-Receptor Interactions and Glial Cell Functions with a Special Focus on G Protein-Coupled Receptors. Int. J. Mol. Sci..

[71] Chvátal A., Syková E. (2000). Glial influence on neuronal signaling. Prog. Brain Res..

[72] Färber K., Kettenmann H. (2005). Physiology of microglial cells. Brain Res. Rev..

[73] Agnati L.F., Marcoli M., Maura G., Woods A., Guidolin D. (2018). The brain as a “hyper-network”: The key role of neural networks as main producers of the integrated brain actions especially via the “broadcasted” neuroconnectomics. J. Neural Transm..

[74] Agnati L.F., Guidolin D., Maura G., Marcoli M., Leo G., Carone C., De Caro R., Genedani S., Borroto-Escuela D.O., Fuxe K. (2014). Information handling by the brain: Proposal of a new “paradigm” involving the roamer type of volume transmission and the tunneling nanotube type of wiring transmission. J. Neural Transm..

[75] Fields R.D., Stevens-Graham B. (2002). New insights into neuron-glia communication. Science.

[76] Kofuji P., Araque A. (2021). G-Protein-Coupled Receptors in Astrocyte-Neuron Communication. Neuroscience.

[77] Sherwood C.C., Stimpson C.D., Raghanti M.A., Wildman D.E., Uddin M., Grossman L.I., Goodman M., Redmond J.C., Bonar C.J., Erwin J.M. (2006). Evolution of increased glia-neuron ratios in the human frontal cortex. Proc. Natl. Acad. Sci. USA.

[78] Von Bartheld C.S., Bahney J., Herculano-Houzel S. (2016). The search for true numbers of neurons and glial cells in the human brain: A review of 150 years of cell counting. J. Comp. Neurol..

[79] Pelassa S., Guidolin D., Venturini A., Averna M., Frumento G., Campanini L., Bernardi R., Cortelli P., Buonaura G.C., Maura G. (2019). A2A-D2 Heteromers on Striatal Astrocytes: Biochemical and Biophysical Evidence. Int. J. Mol. Sci..

[80] Guidolin D., Marcoli M., Tortorella C., Maura G., Agnati L.F. (2020). Adenosine A2A-Dopamine D2 Receptor-Receptor Interaction in Neurons and Astrocytes: Evidence and Perspectives. Prog. Med. Biol. Transl. Sci..

[81] Amato S., Averna M., Guidolin D., Pedrazzi M., Pelassa S., Capraro M., Passalacqua M., Bozzo M., Gatta E., Anderlini D. (2022). Heterodimer of A2A and oxytocin receptors regulating glutamate release in adult striatal astrocytes. Int. J. Mol. Sci..

[82] Sporns O. (2015). Cerebral cartography and connectomics. Philos. Trans. R. Soc. Lond. B Biol. Sci..

[83] Lord L.-D., Stevner A.B., Deco G., Kringelbach M.L. (2017). Understanding principles of integration and segregation using whole-brain computational connectomics: Implications for neuropsychiatric disorders. Phil. Trans. R. Soc. A.

[84] Harvey R.J. (1995). Can computers think? Differences and similarities between computers and brains. Prog. Neurobiol..

[85] Shapshak P. (2018). Artificial Intelligence and brain. Bioinformation.

[86] Singer W., von Braun J., Archer M.S., Reichberg G.M., Sánchez Sorondo M. (2021). Differences Between Natural and Artificial Cognitive Systems. Robotics, AI and Humanity.

[87] El Zaatari S., Marei M., Li W., Usman Z. (2019). Cobot programming for collaborative industrial tasks: An overview. Robot. Auton. Syst..

[88] Guidolin D., Albertin G., Guescini M., Fuxe K., Agnati L.F. (2011). Central nervous system and computation. Q. Rev. Biol..

[89] Lee D. (2020). Birth of Intelligence: From RNA to Artificial Intelligence.

[90] Agnati L.F., Anderlini D., Guidolin DMarcoli M., Maura G. (2022). Man is a “Rope” Stretched Between Virosphere and Humanoid Robots: On the Urgent Need of an Ethical Code for Ecosystem Survival. Found. Sci..

[91] Guidolin D., Marcoli M., Maura G., Agnati L.F. (2017). New dimensions of connectomics and network plasticity in the central nervous system. Rev. Neurosci..

[92] Agnati L.F., Fuxe K., Zoli M., Rondanini C., Ogren S.O. (1982). New vistas on synaptic plasticity: The receptor mosaic hypothesis of the engram. Med. Biol..

[93] Agnati L.F., Guidolin D., Leo G., Fuxe K. (2007). A boolean network modelling of receptor mosaics relevance of topology and cooperativity. J. Neural. Transm..

[94] Dityatev A., Rusakov D.A. (2011). Molecular signals of plasticity at the tetrapartite synapse. Curr. Opin. Neurobiol..

[95] Aggarwal S., Mortensen O.V. (2017). Overview of Monoamine Transporters. Curr. Protoc. Pharmacol..

[96] Fuxe K., Dahlström A.B., Jonsson G., Marcellino D., Guescini M., Dam M., Manger P., Agnati L. (2010). The discovery of central monoamine neurons gave volume transmission to the wired brain. Prog. Neurobiol..

[97] Zhou Y., Danbolt N.C. (2014). Glutamate as a neurotransmitter in the healthy brain. J. Neural. Transm..

[98] Bernardinelli Y., Muller D., Nikonenko I. (2014). Astrocyte-synapse structural plasticity. Neural Plast..

[99] Agnati L.F., Fuxe K., Zoli M., Ozini I., Toffano G., Ferraguti F. (1986). A correlation analysis of the regional distribution of central enkephalin and beta endorphin immunoreactive terminals and of opiate receptors in adult and old male rats. Evidence for the existence of two main types of communication in the central nervous system: The volume transmission and the wiring transmission. Acta Physiol. Scand..

[100] Fuxe K., Agnati L.F. (1991). Volume Transmission in the Brain, Novel Mechanisms for Neural Transmission.

[101] Agnati L.F., Fuxe K. (2000). Volume transmission as a key feature of information handling in the central nervous system: Possible new interpretative value of the Turing’s B-type machine. Progr. Brain Res..

[102] Marcoli M., Agnati L.F., Benedetti F., Genedani S., Guidolin D., Ferraro L., Maura G., Fuxe K. (2015). On the role of the extracellular space on the holistic behavior of the brain. Rev. Neurosci..

[103] Kiss J.P., Vizi E.S. (2001). Nitric oxide: A novel link between synaptic and nonsynaptic transmission. Trends Neurosci..

[104] Agnati L.F., Guidolin D., Guescini M., Genedani S., Fuxe K. (2010). Understanding wiring and volume transmission. Brain Res. Rev..

[105] Taber K.H., Hurley R.A. (2014). Volume transmission in the brain: Beyond the synapse. J. Neuropsychiatry Clin. Neurosci..

[106] Greitz D. (1993). Cerebrospinal fluid circulation and associated intracranial dynamics. A radiologic investigation using MR imaging and radionuclide cisternography. Acta Radiol. Suppl..

[107] Yablonskiy D.A., Ackerman J.J., Raichle M.E. (2000). Coupling between changes in human brain temperature and oxidative metabolism during prolonged visual stimulation. Proc. Natl. Acad. Sci. USA.

[108] Bullock T.H. (1997). Signals and signs in the nervous system: The dynamic anatomy of electrical activity is probably information-rich. Proc. Natl. Acad. Sci. USA.

[109] Poo M.M. (1985). Mobility and localization of proteins in excitable membranes. Annu. Rev. Neurosci..

[110] Isakovic J., Dobbs-Dixon I., Chaudhuri D., Mitrecic D. (2018). Modeling of inhomogeneous electromagnetic fields in the nervous system: A novel paradigm in understanding cell interactions, disease ethiology and therapy. Sci. Rep..

[111] Guescini M., Guidolin D., Vallorani L., Casadei L., Gioacchini A.M., Tibollo P., Battistelli M., Falcieri E., Battistin L., Agnati L.F. (2010). C2C12 myoblasts release micro-vesicles containing mtDNA and proteins involved in signal transduction. Exp. Cell Res..

[112] Venturini A., Passalacqua M., Pelassa S., Pastorino F., Tedesco M., Cortese K., Gagliani M.C., Leo G., Maura G., Guidolin D. (2019). Exosomes from Astrocyte Processes: Signaling to Neurons. Front. Pharmacol..

[113] Guescini M., Leo G., Genedani S., Carone C., Pederzoli F., Ciruela F., Guidolin D., Stocchi V., Mantuano M., Borroto-Escuela D.O. (2012). Microvesicle and tunneling nanotube mediated intercellular transfer of g-protein coupled receptors in cell cultures. Exp. Cell Res..

[114] Venero J.L., Vizuete M.L., Machado A., Cano J. (2001). Aquaporins in the central nervous system. Prog. Neurobiol..

[115] Niermann H., Amiry-Moghaddam M., Holthoff K., Witte O.W., Ottersen O.P. (2001). A novel role of vasopressin in the brain: Modulation of activity-dependent water flux in the neocortex. J. Neurosci..

[116] Sperelakis N. (1997). Cell Physiology.

[117] Jacob F. (1977). Evolution and tinkering. Science.

[118] Kandel E.R., Schwartz J.H., Jessel T.M. (2000). Principles of Neural Science.

[119] McCulloch W.S., Pitts W. (1943). A logical calculus of the ideas immanent in nervous activity. Bull. Math. Biophys..

[120] Woo J., Choi K., Kim S.H., Han K., Choi M. (2021). Characterization of multiscale logic operations in the neural circuits. Front. Biosci..

[121] Agnati L.F., Fuxe K., Zini I., Lenzi P., Hökfelt T. (1980). Aspects on receptor regulation and isoreceptor identification. Med. Biol..

[122] Fuxe K., Agnati L.F., Benfenati F., Cimmino M., Algeri S., Hokfelt T., Mutt V. (1981). Modulation by cholecystokinins of ^3^H-spiroperidol binding in rat striatum: Evidence for increased affinity and reduction in the number of binding sites. Acta Physiol. Scand..

[123] Fuxe K., Agnati L.F., Benfenati F., Celani M., Zini I., Zoli M., Mutt V. (1983). Evidence for the Existence of Receptor—Receptor Interactions in the Central Nervous System. Studies on the Regulation of Monoamine Receptors by Neuropeptides. J. Neural Transm..

[124] Agnati L.F., Guidolin D., Vilardaga J.P., Ciruela F., Fuxe K. (2010). On the expanding terminology in the GPCR field: The meaning of receptor mosaics and receptor heteromers. J. Recept. Signal Transduct. Res..

[125] Agnati L.F., Fuxe K., Ferré S. (2005). How receptor mosaics decode transmitter signals. Possible relevance of cooperativity. Trends Biochem. Sci..

[126] Agnati L.F., Celani M.F., Fuxe K. (1983). Cholecystokinin peptides in vitro modulate the characteristics of the striatal 3H-Npropylnorapomorphine sites. Acta Physiol. Scand..

[127] Agnati L.F., Fuxe K., Benfenati F., Zini I., Hökfelt T. (1983). On the functional role of coexistence of 5-HT and substance P in bulbospinal 5-HT neurons. Substance P reduces affinity and increases density of 3H-5-HT binding sites. Acta Physiol. Scand..

[128] Fuxe K., von Euler G., van der Ploeg I., Fredholm B.B., Agnati L.F. (1989). Pertussis toxin treatment counteracts the cardiovascular effects of neuropeptide Y and clonidine in the awake unrestrained rat. Neurosci. Lett..

[129] Fuxe K., Agnati L.F., Harfstrand A., Zoli M., von Euler G., Grimaldi R., Merlo Pich E., Bjelke B., Eneroth P., Benfenati F. (1990). On the role of neuropeptide Y in information handling in the central nervous system in normal and physio- pathological states. Focus on volume transmission and neuropeptide Y/alpha 2 receptor interactions. Ann. N. Y. Acad. Sci..

[130] Monod J. (1979). Chance and Necessity: An Essay on the Natural Philosophy of Modern Biology.

[131] Fenton A.W. (2008). Allostery: An illustrated definition for the ‘second secret of life’. Trends Biochem. Sci..

[132] Agnati L.F., Tarakanov A.O., Guidolin D. (2005). A simple mathematical model of cooperativity in receptor mosaics based on the “symmetry rule”. BioSystems.

[133] Dehner C., Wells R.D., Bond J.S., Klinman J., Siler Masters B.S. (2018). Non-allosteric proteins. Why do proteins have quaternary structure?. Molecular Life Sciences. An Encyclopedic Reference.

[134] Kenakin T. (2007). Allosteric Theory: Taking Therapeutic Advantage of the Malleable Nature of GPCRs. Curr. Neuropharmacol..

[135] Agnati L.F., Guidolin D., Leo G., Guescini M., Pizzi M., Stocchi V., Spano P.F., Ghidoni R., Ciruela F., Genedani S. (2011). Possible new targets for GPCR modulation: Allosteric interactions, plasma membrane domains, intercellular transfer and epigenetic mechanisms. J. Recept. Signal Transduct. Res..

[136] Nussinov R. (2013). The spatial structure of cell signaling systems. Phys. Biol..

[137] Jordan B.A., Devi L.A. (1999). G-protein-coupled receptor heterodimerization modulates receptor function. Nature.

[138] George S.R., Fan T., Xie Z., Tse R., Tam V., Varghese G., O’Dowd B.F. (2000). Oligomerization of mu- and delta-opioid receptors. Generation of novel functional properties. J. Biol. Chem..

[139] Rashid A.J., So C.H., Kong M.M., Furtak T., El-Ghundi M., Cheng R., O’Dowd B.F., George S.R. (2007). D1-D2 dopamine receptor heterooligomers with unique pharmacology are coupled to rapid activation of Gq/11 in the striatum. Proc. Natl. Acad. Sci. USA.

[140] Agnati L.F., Guidolin D., Cervetto C., Borroto-Escuela D.O., Fuxe K. (2016). Role of iso-receptors in receptor-receptor interactions with a focus on dopamine iso-receptor complexes. Rev. Neurosci..

[141] Agnati L.F., Guidolin D., Fuxe K. (2007). The brain as a system of nested but partially overlapping networks. Heuristic relevance of the model for brain physiology and pathology. J. Neural. Transm..

[142] Genedani S., Carone C., Guidolin D., Filaferro M., Marcellino D., Fuxe K., Agnati L.F. (2010). Differential sensitivity of A2A and especially D2 receptor trafficking to cocaine compared with lipid rafts in cotransfected CHO cell lines. Novel actions of cocaine independent of the DA transporter. J. Mol. Neurosci..

[143] Hillion J., Canals M., Torvinen M., Casado V., Scott R., Terasmaa A., Hansson A., Watson S., Olah M.E., Mallol J. (2002). Coaggregation, cointernalization, and codesensitization of adenosine A2A receptors and dopamine D2 receptors. J. Biol. Chem..

[144] Genedani S., Guidolin D., Leo G., Filaferro M., Torvinen M., Woods A.S., Fuxe K., Ferré S., Agnati L.F. (2005). Computer-assisted image analysis of caveolin-1 involvement in the internalization process of adenosine A2A-dopamine D2 receptor heterodimers. J. Mol. Neurosci..

[145] Mores K.L., Cassell R.J., van Rijn R.M. (2019). Arrestin recruitment and signaling by G protein-coupled receptor heteromers. Neuropharmacology.

[146] Agnati L.F., Guidolin D., Carone C., Dam M., Genedani S., Fuxe K. (2008). Understanding neuronal molecular networks builds on neuronal cellular network architecture. Brain Res. Rev..

[147] Agnati L.F., Fuxe K., Woods A., Genedani S., Guidolin D. (2009). Theoretical considerations on the topological organization of receptor mosaics. Curr. Protein Pept. Sci..

[148] Agnati L.F., Guidolin D., Albertin G., Trivello E., Ciruela F., Genedani S., Tarakanov A., Fuxe K. (2010). An integrated view on the role of receptor mosaics at perisynaptic level: Focus on adenosine A_2A_, dopamine D_2_, cannabinoid CB_1_, and metabotropic glutamate mGlu_5_ receptors. J. Recept. Signal Transduct. Res..

[149] Borroto-Escuela D.O., Brito I., Romero-Fernandez W., Di Palma M., Oflijan J., Skieterska K., Duchou J., Van Craenenbroeck K., Suárez-Boomgaard D., Rivera A. (2014). The G protein-coupled receptor heterodimer network (GPCR-HetNet) and its hub components. Int. J. Mol. Sci..

[150] Sriram K., Insel P.A. (2018). G Protein-Coupled Receptors as Targets for Approved Drugs: How Many Targets and How Many Drugs?. Mol. Pharmacol..

[151] Insel P.A., Sriram K., Gorr M.W., Wiley S.Z., Michkov A., Salmerón C., Chinn A.M. (2019). GPCRomics: An Approach to Discover GPCR Drug Targets. Trends Pharmacol. Sci..

[152] Guo S., Zhao T., Yun Y., Xie X. (2022). Recent progress in assays for GPCR drug discovery. Am. J. Physiol.-Cell Physiol..

[153] Maggio R., Fasciani I., Carli M., Petragnano F., Marampon F., Rossi M., Scarselli M. (2021). Integration and Spatial Organization of Signaling by G Protein-Coupled Receptor Homo- and Heterodimers. Biomolecules.

[154] Reiner A., Levitz J. (2018). Glutamatergic Signaling in the Central Nervous System: Ionotropic and Metabotropic Receptors in Concert. Neuron.

[155] Scheefhals N., MacGillavry H.D. (2018). Functional organization of postsynaptic glutamate receptors. Mol. Cell. Neurosci..

[156] Borroto-Escuela D.O., Carlsson J., Ambrogini P., Narváez M., Wydra K., Tarakanov A.O., Li X., Millón C., Ferraro L., Cuppini R. (2017). Understanding the Role of GPCR Heteroreceptor Complexes in Modulating the Brain Networks in Health and Disease. Front. Cell. Neurosci..

[157] Valle-León M., Callado L.F., Aso E., Cajiao-Manrique M.M., Sahlholm K., López-Cano M., Soler C., Altafaj X., Watanabe M., Ferré S. (2021). Decreased striatal adenosine A_2A_-dopamine D_2_ receptor heteromerization in schizophrenia. Neuropsychopharmacology.

[158] Valle-León M., Casajuana-Martin N., Del Torrent C.L., Argerich J., Gómez-Acero L., Sahlholm K., Ferré S., Pardo L., Ciruela F. (2023). Unique effect of clozapine on adenosine A_2A_-dopamine D_2_ receptor heteromerization. Biomed. Pharmacother..

[159] Fernández-Dueñas V., Gómez-Soler M., Valle-León M., Watanabe M., Ferrer I., Ciruela F. (2019). Revealing Adenosine A_2A_-Dopamine D_2_ Receptor Heteromers in Parkinson’s Disease Post-Mortem Brain through a New AlphaScreen-Based Assay. Int. J. Mol. Sci..

[160] Marcoli M., Agnati L.F., Franco R., Cortelli P., Anderlini D., Guidolin D., Cervetto C., Maura G. (2023). Modulating brain integrative actions as a new perspective on pharmacological approaches to neuropsychiatric diseases. Front. Endocrinol..

[161] Li K., Li J., Zheng J., Qin S. (2019). Reactive Astrocytes in Neurodegenerative Diseases. Aging Dis..

[162] Cragnolini A.B., Lampitella G., Virtuoso A., Viscovo I., Panetsos F., Papa M., Cirillo G. (2020). Regional brain susceptibility to neurodegeneration: What is the role of glial cells?. Neural Regen. Res..

[163] Diderot D. (1830). Le Reve de d’Alembert.

[164] Guidolin D., Anderlini D., Maura G., Marcoli M., Cortelli P., Calandra-Buonaura G., Woods A.S., Agnati L.F. (2019). A New Integrative Theory of Brain-Body-Ecosystem Medicine: From the Hippocratic Holistic View of Medicine to Our Modern Society. Int. J. Environ. Res. Public Health.

[165] Quigley E.M.M. (2017). Microbiota-Brain-Gut Axis and Neurodegenerative Diseases. Curr. Neurol. Neurosci. Rep..

[166] Adak A., Khan M.R. (2019). An insight into gut microbiota and its functionalities. Cell. Mol. Life. Sci..

[167] Shen Y., Xu J., Li Z., Huang Y., Yuan Y., Wang J., Zhang M., Hu S., Liang Y. (2018). Analysis of gut microbiota diversity and auxiliary diagnosis as a biomarker in patients with schizophrenia: A cross-sectional study. Schizophr. Res..

[168] Calvani R., Picca A., Lo Monaco M.R., Landi F., Bernabei R., Marzetti E. (2018). Of Microbes and Minds: A Narrative Review on the Second Brain Aging. Front. Med..

[169] Fülling C., Dinan T.G., Cryan J.F. (2019). Gut Microbe to Brain Signaling: What Happens in Vagus…. Neuron.

[170] Fil J., Dalchau N., Chu D. (2022). Programming Molecular Systems to Emulate a Learning Spiking Neuron. ACS Synth. Biol..

[171] Agnati L.F., Ferré S., Genedani S., Leo G., Guidolin D., Filaferro M., Carriba P., Casado V., Lluis C., Franco R. (2006). Allosteric Modulation of Dopamine D_2_ Receptors by Homocysteine. J. Proteome Res..

[172] Nussinov R., Tsai C.-J., Liu J. (2014). Principles of Allosteric Interactions in Cell Signaling. J. Am. Chem. Soc..

[173] Schiffer F. (2019). The physical nature of subjective experience and its interaction with the Brain. Med. Hypotheses.

[174] Friston K.J. (2019). Waves of prediction. PLoS Biol..

[175] Feynman R.P. (1998). The Meaning of It All Thoughts of a Citizen Scientist.

[176] Dickinson E., Johnson T.H. (1890). J632 (1862). The Complete Poems of Emily Dickinson.

